# TLR7 Agonist-Loaded Gadolinium Oxide Nanotubes Promote Anti-Tumor Immunity by Activation of Innate and Adaptive Immune Responses

**DOI:** 10.3390/vaccines12040373

**Published:** 2024-04-01

**Authors:** Xiupeng Wang, Motohiro Hirose, Xia Li

**Affiliations:** Health and Medical Research Institute, Department of Life Science and Biotechnology, National Institute of Advanced Industrial Science and Technology (AIST), Central 6, 1-1-1 Higashi, Tsukuba 305-8566, Japan; motohiro-hirose@aist.go.jp (M.H.); li.xia@nims.go.jp (X.L.)

**Keywords:** Gd_2_O_3_ nanotubes, adjuvant, cancer, immunotherapy

## Abstract

Improving the delivery of biomolecules to DCs and lymph nodes is critical to increasing their anti-tumor efficacy, reducing their off-target side effects, and improving their safety. In this study, Gd_2_O_3_ nanotubes with lengths of 70–80 nm, diameters of 20–30 nm, and pore sizes of up to 18 nm were synthesized using a facile one-pot solvothermal method. The Gd_2_O_3_ nanotubes showed good adsorption capacity of OVA and TLR7a, with a loading efficiency of about 100%. The Gd_2_O_3_ nanotubes showed pH-sensitive degradation and biomolecule release properties; the release of gadolinium ions, OVA, and TLR7a was slow at pH 7.4 and fast at pH 5. The Gd_2_O_3_ nanotubes showed 2.6–6.0 times higher payload retention around the injection site, 3.1 times higher cellular uptake, 1.7 times higher IL1β secretion, 1.4 times higher TNFα secretion by BMDCs, and markedly enhanced draining lymph node delivery properties. The combination of OVA, TLR7a, and Gd_2_O_3_ nanotubes significantly inhibited tumor growth and increased survival rate compared with only OVA-TLR7a, only OVA, and saline. The Gd_2_O_3_ nanotubes are biocompatible and can also be used as radiation sensitizers.

## 1. Introduction

Cancer immunotherapy has revolutionized cancer treatment owing to recent clinical advances in cancer immunotherapy drug development [1,2]. However, tumors can evade immune system attack by evading immune cell recognition, secreting immunosuppressive cytokines, expressing immune checkpoints, producing regulatory T lymphocytes (Treg), inducing the apoptosis of immune cells, and generating M2 macrophages [3,4]. To boost host immune responses against cancer, a promising strategy involves the isolation and activation of dendritic cells (DCs) ex vivo, followed by the injection of these activated DCs back into patients. After homing to lymph nodes, the activated DCs present the loaded cancer antigens to T cells to initiate anti-tumor immune responses [5,6]. However, this strategy has disadvantages. It requires a long in vitro pretreatment time and only a small fraction of programmed DCs can be delivered to lymph nodes. Moreover, it is costly and there are difficulties with quality control and regulatory concerns [5,6]. Therefore, it is important to complete DC recruitment and activation, antigen presentation, and T cell activation directly inside the body [7].

The toll-like receptor 7 agonist (TLR7a) is a promising candidate that can enhance the innate and adaptive immune responses through the expression of CD40, CD80, CD86, and CCR7, and the secretion of interleukin (IL)6, IL8, IL12, interferon (IFN)α, IFNγ, and tumor necrosis factor α (TNFα) [8,9]. Imiquimod is an imidazoquinoline amine that shows immune modulating and anti-tumor effects by binding to TLR7, mainly expressed by plasmacytoid DCs, macrophages, mast cells, and monocytes [10,11]. Imiquimod is an FDA-approved molecular immunopotentiator for topical application for the treatment of skin malignancies [10,11]. Imiquimod activates TLR7 through an MyD88-dependent signaling pathway and induces the expression of the transcription factor NF-κB [12,13]. However, the systemic application of imiquimod showed a limited success in clinical trials because of an imbalance between safety and efficacy [9,10,14]. Its fast clearance from the injection site, limited uptake efficiency by antigen-presenting cells, and inefficient delivery to draining lymph nodes necessitate high doses, thus causing off-target side effects and systemic toxicity [9,10,14]. Nanoparticle-based adjuvants are critical for overcoming the shortcomings of soluble immunopotentiators, which extend the retention time, increase the uptake efficiency by antigenpresenting cells, and increase delivery efficiency to draining lymph nodes [15,16,17,18,19].

Nanoparticles formulated from polymers, lipids, and inorganic materials have shown success in enabling a more efficient and sustained delivery of the loaded components to the target cells and tissues [15,20,21]. Inorganic materials can protect against payload degradation, extend payload exposure, facilitate payload uptake by DCs, improve payload bioavailability, reduce the necessary payload dose, and control the induced immune response [15,16,17,18,19]. In particular, gadolinium-based substances are commonly used as contrast agents in clinical magnetic resonance imaging, which are proved to be safe for humans [22,23]. Herein, we synthesized a new type of Gd_2_O_3_ nanotube. We hypothesized that the encapsulation of TLR7a with Gd_2_O_3_ nanotubes would overcome the drawback of free TLR7a and significantly improve payload delivery to antigen-presenting cells (APCs), thereby ultimately increasing the anti-tumor efficacy.

## 2. Materials and Methods

### 2.1. Synthesis of Gd_2_O_3_ Nanotubes

Gadolinium acetate hydrate and urea were dissolved in ethanol (FUJIFILM Wako Pure Chemical Corporation, Tokyo, Japan) by stirring at room temperature to form a clear solution (molar ratio of gadolinium acetate hydrate, urea, and ethanol = 1:2:206). Next, the solution was heated at 160 °C for 9 h in a polytetrafluoroethylene tube. The precipitates were washed with ultrapure water and freeze-dried to obtain Gd_2_O_3_ nanotubes.

### 2.2. Characterization of Gd_2_O_3_ Nanotubes

The Gd_2_O_3_ nanotubes were characterized by transmission electron microscopy (TEM, JEOL, Tokyo, Japan) and powder X-ray diffraction (XRD) analysis (Rigaku, Tokyo, Japan). The nitrogen gas (N_2_) adsorption–desorption isotherm of the Gd_2_O_3_ nanotubes was characterized using a surface area and porosity analyzer (TriStar II, Micromeritics, Norcross, GA, USA).

### 2.3. Biomolecule Loading and Release, and Gd_2_O_3_ Nanotube Degradation In Vitro

Chicken egg ovalbumin (OVA, Sigma-Aldrich, St. Louis, MO, USA, 1 mg/mL in saline) and TLR7a (imiquimod, InVivoGen, San Diego, CA, USA, 0.2 mg/mL in saline) were mixed with Gd_2_O_3_ nanotubes (10 mg/mL) at 4 °C for 1 day. The supernatants were collected by centrifugation. The remaining OVA in the supernatant was tested by Bio-Rad Protein Assay (Bio-Rad Laboratories, Inc., Hercules, CA, USA). The remaining TLR7a in the supernatant was tested by an ultraviolet–visible spectrophotometer (V-550, JASCO, Tokyo, Japan). The loading efficiencies of OVA and TLR7a were calculated by the following formula: loading efficiency = (Initial biomolecule concentration−Biomolecule concentration after loading)/Initial biomolecule concentration × 100%.

To examine the biomolecule release and Gd_2_O_3_ nanotube degradation, the biomolecule-loaded Gd_2_O_3_ nanotubes (2.5 mg) were added to the acetate buffer (2 mL, pH = 5) or the Tris-HCl buffer (2 mL, pH = 7.4) at 37 °C. At certain time intervals, the buffers were collected, and at the same time, 1 mL of fresh buffers was added. The collected buffers were analyzed for OVA and TLR7a concentrations. The Gd ion concentrations in the collected buffers were analyzed by inductively coupled plasma-atomic emission spectrometry (ICP-AES, Hitachi High-Technologies, Ibaraki, Japan).

### 2.4. In Vivo Antigen Retention

C57BL/6J mice (female, 6 weeks old, CLEA Inc., Tokyo, Japan) were subcutaneously injected with Alexa Fluor 647-OVA (A647-OVA, Molecular Probes, Eugene, OR, USA, 100 μg/mouse) and Gd_2_O_3_-A647-OVA (100 μg/mouse for A647-OVA; 1 mg/mouse for Gd_2_O_3_ nanotubes). The distribution of A647-OVA in mice was analyzed using an in vivo imaging system (IVIS).

### 2.5. In Vitro Cellular Test

Bone marrow-derived dendritic cells (BMDCs) collected in accordance with a previous report [24] were used for in vitro testing. At first, Gd_2_O_3_ nanotubes were mixed with a green fluorescent fluorescein ovalbumin conjugate (F-OVA, Life Technologies, Carlsbad, CA, USA) at 4 °C overnight. Then, BMDCs were cultured with F-OVA and F-OVA-loaded Gd_2_O_3_ nanotubes (25 μg/mL for Gd_2_O_3_ nanotubes; 5 μg/mL for F-OVA). After culture for 1 d, the BMDCs were washed with calcium- and magnesium-free phosphate buffered saline [PBS(-)] and tested using a fluorescent microplate reader (MTP-900, Hitachi). In addition, the cells were stained with LysoTracker red DND-99 (Invitrogen, Waltham, MA, USA) and Hoechst (Thermo Fisher, Waltham, MA, USA), and analyzed using a confocal laser scanning microscope (Leica, Wetzlar, Germany, TCS SP5). The media were also collected after culture for 2 days and tested using mouse TNFα and IL1β enzyme-linked immunosorbent assay (ELISA) kits (BD Biosciences, San Jose, CA, USA).

### 2.6. In Vivo Antigen Delivery in Lymph Nodes and In Vivo Safety

C57BL/6J mice (female, 6 weeks old, CLEA Inc.) were subcutaneously injected with F-OVA (100 μg/mouse) and Gd_2_O_3_-F-OVA (100 μg/mouse for F-OVA; 1 mg/mouse for Gd_2_O_3_ nanotubes). Cells around the injection site were collected after 16 h and analyzed by flow cytometry (FACSAria, BD Bioscience). Nearby draining lymph nodes were collected to prepare cryosections, stained with DAPI (Funakoshi, Tokyo, Japan) and observed under a fluorescence microscope (BX51, Olympus, Tokyo, Japan) equipped with a highly sensitive camera (DP74, Olympus). The kidney, spleen, heart, liver, and lung were collected 16 h after injection, and stained with hematoxylin, and eosin (HE). Blood urea nitrogen (BUN), aspartate aminotransferase (AST), creatinine (CRE), alanine aminotransferase (ALT), and alkaline phosphatase (ALP) levels (Sysmex, Hyogo, Japan) of mice 2 d after subcutaneous administration of saline and Gd_2_O_3_ were tested.

### 2.7. In Vivo Anti-Tumor Immunity

E.G7-OVA cells (2 × 10^5^ cells/mouse, ATCC, Manassas, VA, USA) were injected subcutaneously into the right flank of C57BL/6 mice (6 weeks old, female, CLEA Inc.). On days 7, 10, 14, and 21, mice were divided into 4 groups, and saline, OVA (100 μg/mouse), OVA-TLR7a (100 μg/mouse for OVA; 20 μg/mouse for Imiquimod), or Gd_2_O_3_-OVA-TLR7a (100 μg/mouse for OVA; 20 μg/mouse for Imiquimod; 1 mg/mouse for Gd_2_O_3_ nanotubes) were injected into their left flanks. The tumor volume was calculated by 1/2 × longest dimension × (perpendicular dimension)^2^. Mouse survival rate was calculated on the basis of tumor size < 15 mm. Splenocytes were collected, stained with anti-mouse CD4, anti-mouse CD8α, anti-mouse IFNγ, and anti-mouse TNFα antibodies (BioLegend, San Diego, CA, USA), and analyzed using a FACSAria cell cytometer (BD Biosciences).

### 2.8. Radiation Sensitization Evaluation of Gd_2_O_3_ Nanotubes

E.G7-OVA cells and mouse oral squamous cell carcinoma 2 (MOC2) cells (Kerafast, Boston, MA, USA) (5 × 10^3^ cells/well) were seeded on 96-well plates and cultured overnight. Gd_2_O_3_ nanotubes (0–50 μg/mL for E.G7-OVA; 0–200 μg/mL for MOC2) were added and cultured for 4 h. The cells were exposed to radiation (0–2 Gy for E.G7-OVA; 0–8 Gy for MOC2) using an X-ray generator (Faxitron X-ray Corp., Lincolnshire, IL, USA, CP160), and then cultured for another 3 days. Cell viability was determined using a CCK-8 kit (Dojindo Molecular Technologies, Rockville, MD, USA).

MOC2 cells (2.5 × 10^4^ cells/well) were seeded on 96-well plates and cultured for 24 h. Gd_2_O_3_ nanotubes (200 μg/mL) were added and cultured for another 6 h. The cells were washed with PBS (-) and cultured with DCFDA (30 μmol) for 45 min. The ROS generation capacity was analyzed using a DCFDA/H2DCFDA-cellular ROS assay kit (Abcam, Cambridge, UK).

MOC2 cells (2.5 × 10^5^ cells/well) were seeded on 12-well plates and cultured overnight. Gd_2_O_3_ nanotubes (200 μg/mL) were added and the cells were further cultured overnight. The cells were exposed to radiation at a dose of 6 Gy using an X-ray generator. Then, the cells were analyzed using a DNA damage detection kit-γ-H2AX-Green (Dojindo).

### 2.9. Statistical Analysis

Log-rank test, Student’s *t*-test, or ANOVA with Tukey’s multiple comparisons post hoc test were used to calculate the statistical significance of differences. A *p*-value of <0.05 was considered statistically significant.

## 3. Results and Discussion

**Figure 1:**
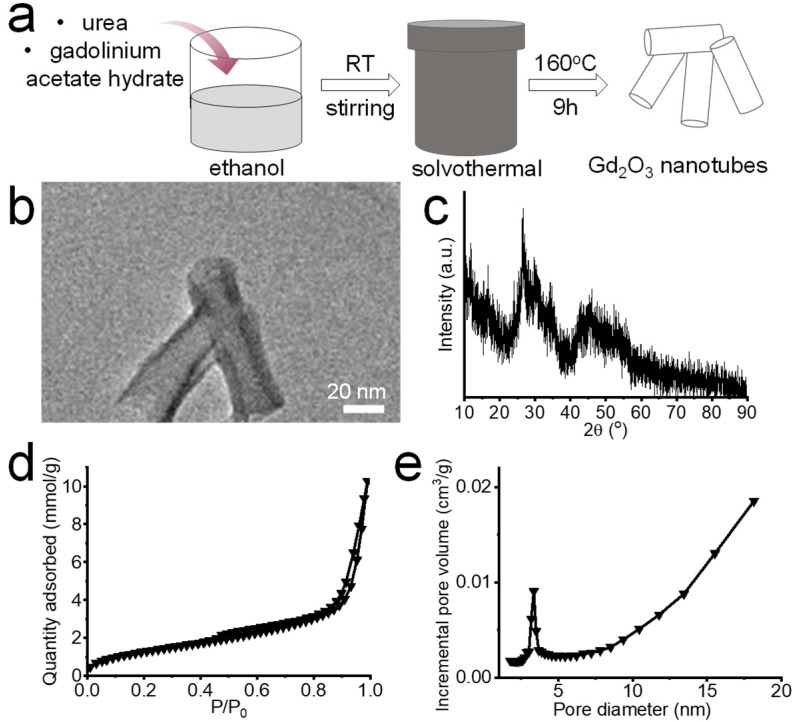
Synthesis scheme of Gd_2_O_3_ nanotubes (**a**), TEM image (**b**), XRD pattern (**c**), N_2_ adsorption-desorption isotherms, (**d**) and pore size distribution (**e**) of Gd_2_O_3_ nanotubes.

Gd_2_O_3_ nanotubes were synthesized by a facile one-pot solvothermal method using gadolinium acetate hydrate, urea, and ethanol (Figure 1a). The Gd_2_O_3_ nanotubes were about 70–80 nm in length and 20–30 nm in diameter (Figure 1b), and were composed of amorphous Gd_2_O_3_ without extra peaks caused by impurity phases, as shown by the XRD pattern in Figure 1c. Pore sizes of up to 18 nm were observed on the pore size distribution curve of Gd_2_O_3_ nanotubes (Figure 1d,e), which was consistent with the TEM image in Figure 1b.

**Figure 2:**
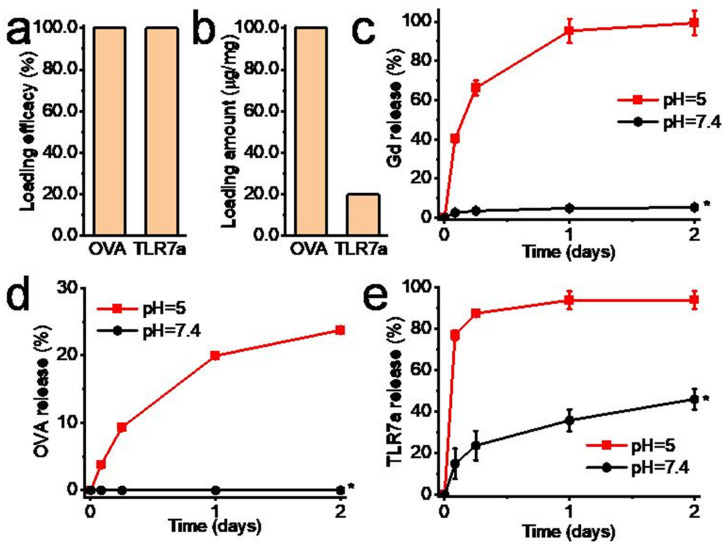
Biomolecule loading and release, and Gd_2_O_3_ nanotube degradation. OVA and TLR7a loading efficiency (**a**) and loading amount (**b**) on Gd_2_O_3_ nanotubes, pH-responsive Gd_2_O_3_ nanotube degradation (**c**), pH-responsive OVA, (**d**) and TLR7a (**e**) release from Gd_2_O_3_-OVA and Gd_2_O_3_-TLR7a, respectively (n = 3–4, * *p* < 0.05).

The Gd_2_O_3_ nanotubes showed good adsorption capacity of OVA and TLR7a, with a loading efficiency of about 100% (Figure 2a). The amounts of OVA and TLR7a loaded to the Gd_2_O_3_ nanotubes were about 100 μg/mg and 20 μg/mg, respectively (Figure 2b). The Gd_2_O_3_ nanotubes showed pH-sensitive degradation and biomolecule release from Gd_2_O_3_-OVA and Gd_2_O_3_-TLR7a. Gadolinium ions, OVA, and TLR7a were released slowly in a slightly alkaline Tris-HCl buffer (pH = 7.4). In contrast, gadolinium ions, OVA, and TLR7a were released rapidly in an acidic acetate buffer (pH = 5). In the acidic acetate buffer, the release percentages of gadolinium ions, OVA, and TLR7a reached 99%, 24%, and 91% on day 2, respectively (Figure 2c–e). Gd_2_O_3_ nanotubes showed pH-sensitive degradation manner, with a faster degradation rate at pH 5 and a slower degradation rate at pH 7.4 (Figure 2c). Therefore, OVA and TLR7a loaded with Gd_2_O_3_ nanotubes showed a pH-sensitive release manner similar to the degradation manner of Gd_2_O_3_ nanotubes. The slow release of biomolecules in a slightly alkaline environment and the rapid release of biomolecules in an acidic environment is beneficial for reducing the delivery of biomolecules into the extracellular environment and promoting the biomolecule delivery into the intracellular environment [25].

**Figure 3:**
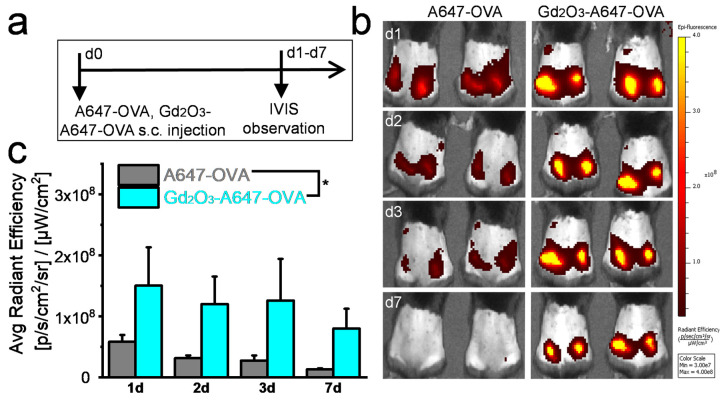
Gd_2_O_3_ nanotubes significantly prolonged A647-OVA retention around the injection site. Experimental procedure (**a**), IVIS images of A647-OVA in mice (**b**), radiant efficiency around injection site, and (**c**) 1, 2, 3 and 7 d after subcutaneous injection (n = 4, * *p* < 0.05).

A647-OVA was used to evaluate the in vivo biomolecule retention around the injection site (Figure 3a). For mice injected with free A647-OVA, the signal intensity of A647-OVA decreased rapidly from d1 to the detection limit on d7. For mice injected with Gd_2_O_3_-A647-OVA, the signal intensity of A647-OVA decreased much more slowly than those injected with free A647-OVA (Figure 3b). From d1 to d7, mice injected with Gd_2_O_3_-A647-OVA showed a 2.6–6.0 times higher A647-OVA signal intensity than those injected with free A647-OVA (Figure 3c). The prolonged release of biomolecules is essential for the long-term stimulation of DCs to break immune tolerance. The rapid removal of biomolecules may reduce the duration and quality of the generated immune memory [20,26]. In this study, the Gd_2_O_3_ nanotubes showed a significantly prolonged payload release in vivo, so they are favorable for initiating robust and long-term adaptive immune responses [27,28].

**Figure 4:**
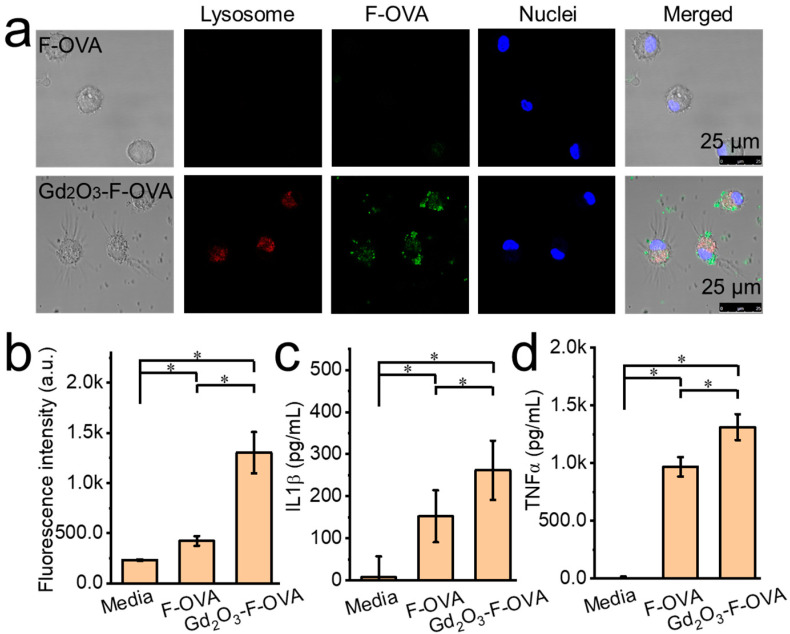
Gd_2_O_3_ nanotubes facilitated F-OVA cellular uptake and BMDC maturation in vitro. Representative confocal laser scanning microscope images of F-OVA and Gd_2_O_3_-F-OVA after culture with BMDCs with lysosome staining (**a**), fluorescence intensity of F-OVA after cellular uptake by BMDCs (**b**), IL1β (**c**), and TNFα (**d**) secretion levels from BMDCs (n = 6, * *p* < 0.05).

The Gd_2_O_3_ nanotubes markedly facilitated antigen cellular uptake and the maturation of BMDCs in vitro. BMDCs engulfed only a small amount of free F-OVA, as shown by the very weak fluorescence signals in the representative confocal laser scanning microscopy images. In contrast, BMDCs engulfed markedly large amounts of F-OVA from Gd_2_O_3_-F-OVA, in which strong fluorescence signals were detected in representative confocal laser scanning microscopy images (Figure 4a). The fluorescence intensity of BMDCs cultured with Gd_2_O_3_-F-OVA was 3.1 times higher than those cultured with F-OVA (Figure 4b). The Gd_2_O_3_ nanotubes increased levels of IL1β and TNFα secretion from BMDCs. BMDCs cultured with Gd_2_O_3_-F-OVA showed 1.7 and 1.4 times higher IL1β and TNFα secretion levels than those cultured with F-OVA (Figure 4c,d). BMDCs treated with Gd_2_O_3_-OVA showed the highest CD86^+^, CCR7^+^, and CD80^+^ expression among BMDCs treated with unloaded Gd_2_O_3_ nanotubes and culture media. BMDCs treated with unloaded Gd_2_O_3_ nanotubes showed comparable expressions of CD86^+^, CCR7^+^, and CD80^+^ compared with BMDCs treated with culture media (Figure S1). The activation of cytotoxic T lymphocytes (CTLs) is critical for anti-tumor immunity, which requires the delivery of antigens to DCs, the activation of DCs, the cross-presentation of antigens to T cells, and the activation of T cells [29]. DCs recognize pathogen components, up-regulate costimulatory molecules, secrete cytokines and chemokines, promote antigen cross-presentation, and induce subsequent immune responses [21,30,31,32].

**Figure 5:**
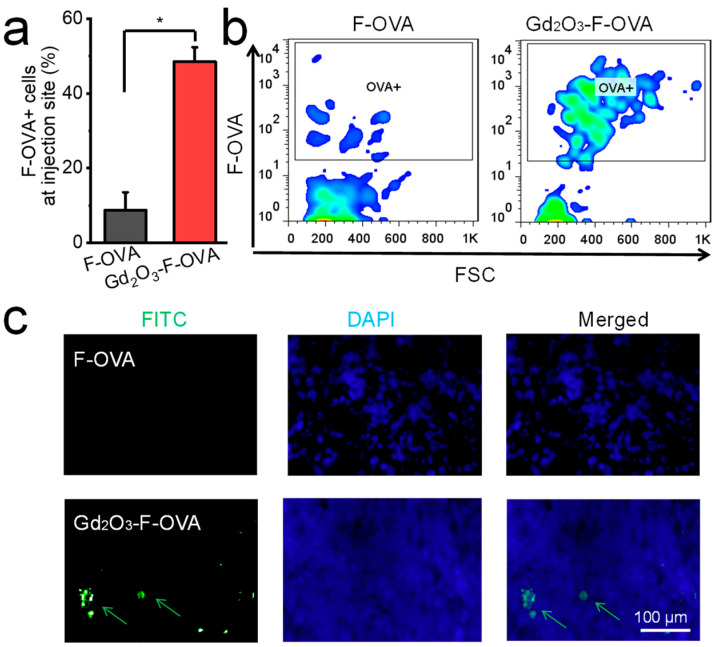
Gd_2_O_3_ nanotubes facilitated F-OVA cellular uptake and delivery of F-OVA into the lymph nodes. F-OVA positive cells at the injection site (**a**,**b**; n = 3, * *p* < 0.05), and representative cryosection images of mouse lymph nodes 16 h after subcutaneous injection of F-OVA and Gd_2_O_3_-F-OVA (**c**).

The Gd_2_O_3_ nanotubes increased the F-OVA cellular uptake efficiency in vivo. The percentages of F-OVA^+^ cells were 9% and 49% around injection site in mice 16 h after injection with F-OVA and Gd_2_O_3_-F-OVA, respectively (Figure 5a,b). The Gd_2_O_3_ nanotubes facilitated the delivery of antigens into nearby draining lymph nodes. Only weak antigen signals were detected in the nearby draining lymph nodes of mice injected with F-OVA. In contrast, higher antigen signal intensities were detected in the nearby draining lymph nodes of mice injected with Gd_2_O_3_-F-OVA than in those of mice injected with free F-OVA 16 h after subcutaneous injection (Figure 5c). Recent studies have demonstrated that the targeted delivery of immune potentiators is superior to the administration of their free forms. The delivery efficiency of immune potentiators to APCs and lymphoid tissues determines the quality of subsequent immune responses [9,21,33]. In addition, the systemic circulation of immune potentiators may cause undesired toxicity [34]. Therefore, the Gd_2_O_3_ nanotubes that promote the uptake of immune potentiators by DCs and their transport to lymphoid tissues are critical for improving immunogenicity and reducing the off-target morbidity of immune potentiators.

**Figure 6:**
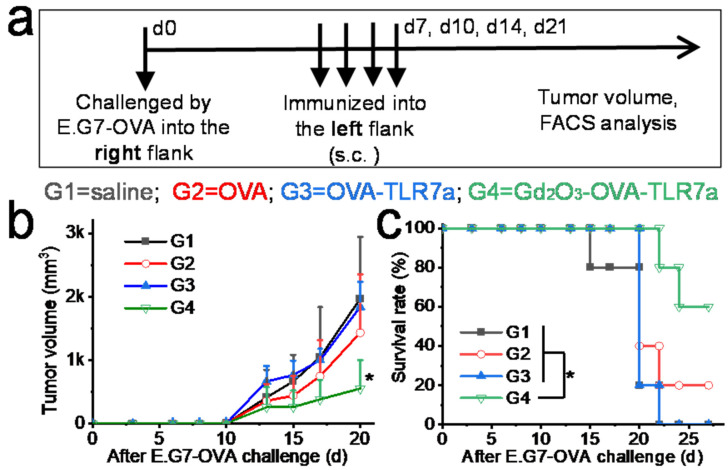
Gd_2_O_3_ nanotubes promoted anti-tumor immunity in mice. Experimental procedure (**a**), tumor volume (**b**), and survival rate (**c**) of mice administrated with G1: saline, G2: OVA, G3: OVA-TLR7a, and G4: Gd_2_O_3_-OVA-TLR7a (n = 5, * *p* < 0.05).

The combination of OVA, TLR7a, and Gd_2_O_3_ nanotubes significantly inhibited tumor growth and increased survival rate compared with saline, only OVA, and OVA-TLR7a in a therapeutic mouse model. The tumor volumes of the mice injected with saline, only OVA, OVA-TLR7a, and Gd_2_O_3_-OVA-TLR7a were 1970 ± 973 mm^3^, 1435 ± 919 mm^3^, 1839 ± 397 mm^3^, and 556 ± 445 mm^3^ on d20, respectively (Figure 6a,b). The survival rates of the mice injected with saline, only OVA, OVA-TLR7a, and Gd_2_O_3_-OVA-TLR7a were 0%, 20%, 0%, and 60% at the endpoint, respectively (Figure 6c). The anti-tumor effect of Gd_2_O_3_ alone should be further studied.

**Figure 7:**
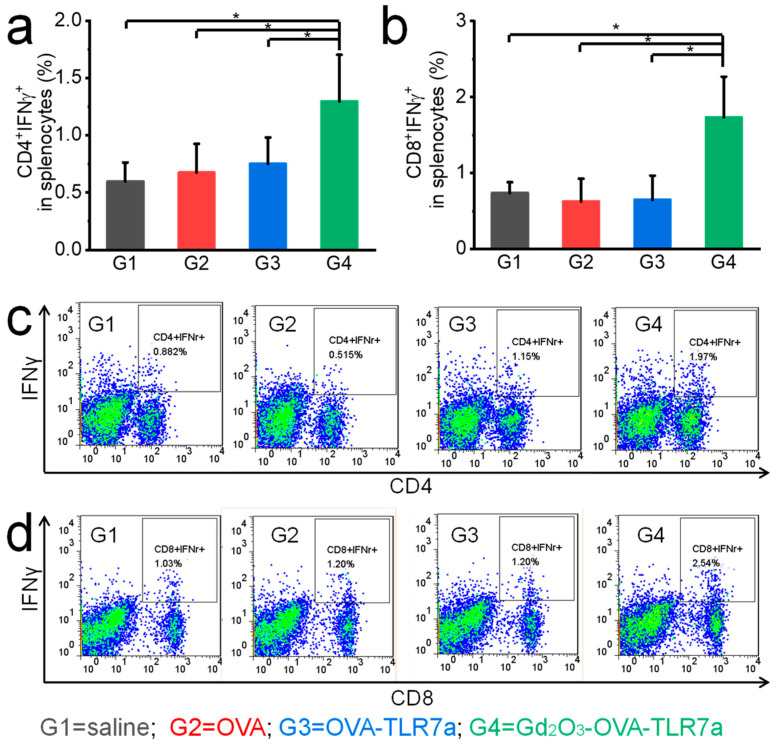
Gd_2_O_3_ nanotubes promoted IFNγ expression in splenocytes of mice. CD4^+^IFNγ^+^ T cell populations (**a**) and CD8^+^IFNγ^+^ T cell populations (**b**), representative flow cytometry plots of CD4^+^IFNγ^+^ T cells (**c**) and CD8^+^IFNγ^+^ T cells (**d**) at the endpoint (n = 5, * *p* < 0.05).

**Figure 8:**
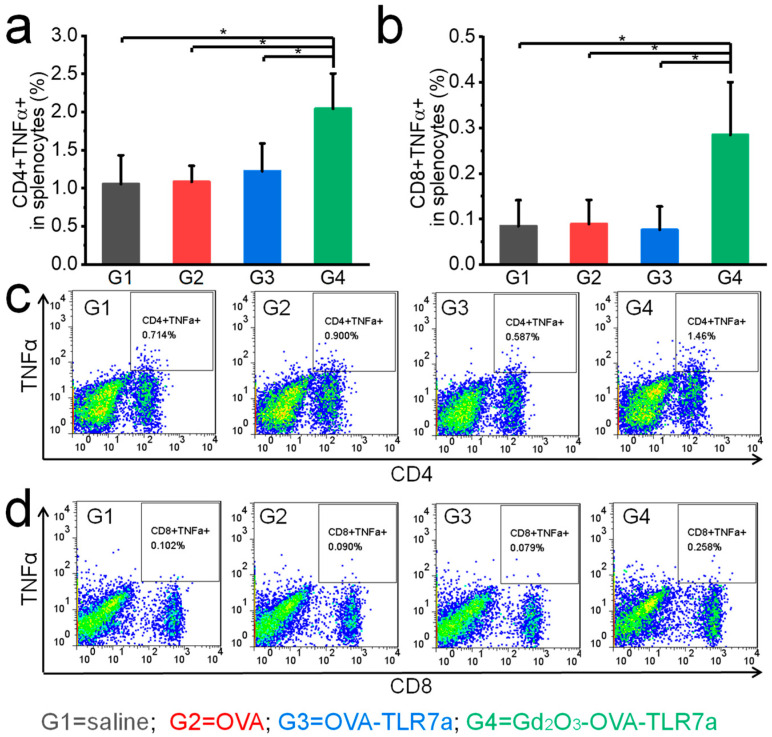
Gd_2_O_3_ nanotubes promoted TNFα expression in splenocytes of mice. CD4^+^TNFα^+^ T cell populations (**a**) and CD8^+^TNFα^+^ T cell populations (**b**), representative flow cytometry plots of CD4^+^TNFα^+^ T cells (**c**), and CD8^+^TNFα^+^ T cells (**d**) at the endpoint (n = 5, * *p* < 0.05).

The combination of OVA, TLR7a, and Gd_2_O_3_ nanotubes significantly increased IFNγ and TNFα expression levels in splenocytes of mice at the endpoint (Figure 7 and Figure 8). In splenocytes of the mice injected with saline, only OVA, OVA-TLR7a, and Gd_2_O_3_-OVA-TLR7a showed CD4^+^IFNγ^+^ T cell populations of 0.60%, 0.67%, 0.75%, and 1.30% (Figure 7); CD8^+^IFNγ^+^ T cell populations of 0.74%, 0.63%, 0.65%, and 1.74% (Figure 7); CD4^+^TNFα^+^ T cell populations of 1.06%, 1.08%, 1.22%, and 2.04% (Figure 8); and CD8^+^TNFα^+^ T cell populations of 0.08%, 0.09%, 0.08%, and 0.29% (Figure 8), respectively.

**Figure 9:**
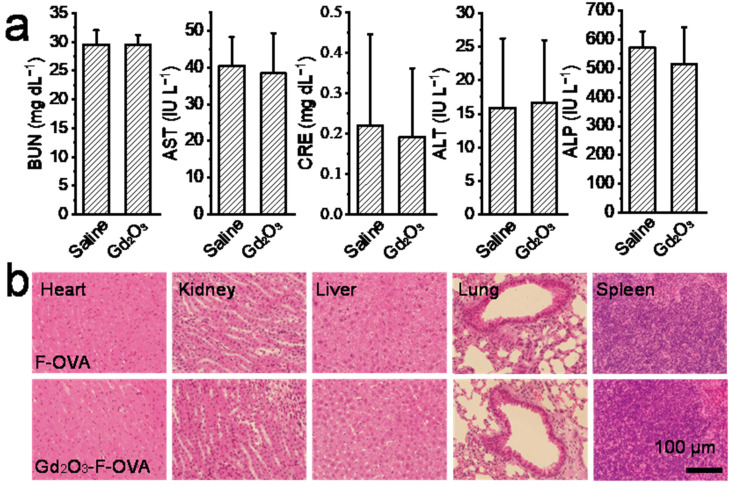
Biocompatibility of Gd_2_O_3_ nanotubes. Biochemistry parameters (BUN, AST, CRE, ALT, and ALP) of mice 2d after subcutaneously injection of saline and Gd_2_O_3_ (**a**, n = 3), and histological sections of heart, kidney, liver, lung, and spleen of mice 16 h after subcutaneously administration of F-OVA and Gd_2_O_3_-F-OVA (**b**).

Several clinical studies have shown that cancer vaccines can induce protection in prophylactic mouse models; however, the same vaccines often show only limited efficacy in therapeutic mouse models [35]. To generate anti-tumor immune responses, robust and durable T cell activation plays a central role, because they kill tumor cells and secrete tumor suppressive cytokines [36,37]. Among the immune potentiators, TLRa shows promising potential in activating T cells followed by APC activation, antigen presentation, and cytokine secretion [38]. TLRa stimulates a variety of APCs, especially DCs, to present antigens, express costimulatory molecules, secrete cytokines, and initiate T cell responses [9,38]. The efficacy of TLRa can be further improved by using delivery systems that can overcome the restricted TLRa distribution, and prolonged activity in draining lymph nodes, thus improving immunogenicity and reducing systemic distribution and morbidity [9]. In this study, the combination of OVA, TLR7a, and Gd_2_O_3_ nanotubes significantly inhibited tumor growth in the therapeutic mouse model by increasing IFNγ and TNFα expression levels in splenocytes of mice at the endpoint (Figure 7, Figure 8 and Figure 9). IFNγ and TNFα play pivotal roles in the activation and stimulation of anti-tumor immune responses [39,40,41,42]. IFNγ may inhibit tumor angiogenesis, induce regulatory T cell apoptosis, stimulate M1 macrophage activity, and promote anti-tumor immunity [39,40]. TNFα, a potent paracrine and endocrine mediator of inflammation and immunity, plays a key role in regulating the growth and differentiation of various cells, as well as anti-tumor activity [41,42].

The Gd_2_O_3_ nanotubes are biocompatible as shown by the blood biochemical tests and histological analysis (Figure 9). The BUN, AST, CRE, ALT, and ALP levels in mice injected with Gd_2_O_3_ nanotubes and saline were within normal ranges, with no significant differences (Figure 9a). Moreover, there was no marked difference in histological sections of the heart, kidneys, liver, lungs, and spleen of mice injected with Gd_2_O_3_-F-OVA and F-OVA, indicating no obvious toxicity of Gd_2_O_3_ nanotubes (Figure 9b).

**Figure 10:**
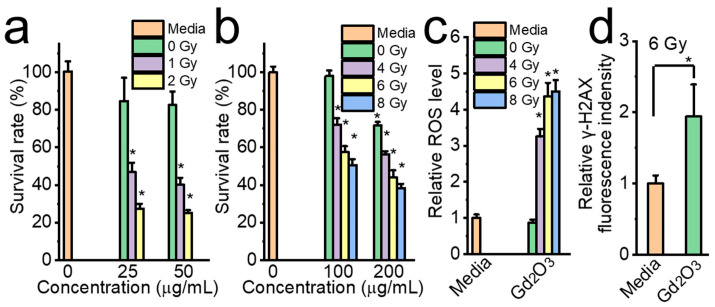
Together with irradiation, Gd_2_O_3_ nanotubes decreased survival rate of E.G7-OVA and MOC2, increased intracellular ROS generation and intracellular DNA damage of MOC2. Survival rate of E.G7-OVA (**a**, n = 8) and MOC2 (**b**, n = 8), relative ROS level (**c**, n = 8), and relative γ-H2AX fluorescence intensity (**d**, n = 4, * *p* < 0.05).

In addition to the immune stimulating effect, the Gd_2_O_3_ nanotubes also work as radiation sensitizers. The Gd_2_O_3_ nanotubes markedly decrease survival rate, increase ROS levels, and enhance DNA damage when used together with radiation. The survival rates of E.G7-OVA and MOC2 cells decreased with increasing Gd_2_O_3_ concentration and radiation dose (Figure 10a,b). The survival rate of E.G7-OVA cells decreased to 25% in the case of 50 μg/mL Gd_2_O_3_ and 2 Gy radiation (Figure 10a). The survival of MOC2 cells decreased to 38% with 200 μg/mL Gd_2_O_3_ and 8 Gy radiation (Figure 10b). MOC2 cells treated with 200 μg/mL Gd_2_O_3_ and 6 Gy radiation showed 4.4 times and 1.9 times higher ROS levels and relative γ-H2AX fluorescence intensities than those without any treatment, respectively (Figure 10c,d). Although animal and clinical trials have indicated the feasibility of personalized cancer vaccines composed of peptides and RNA [43], their high development cost and long development period have hampered the clinical application [44]. The combination of Gd_2_O_3_ nanotubes with radiotherapy is advantageous for inducing tumor cell death, after which the adsorption of dead tumor components on Gd_2_O_3_ nanotubes may lead to the formation of an in situ cancer vaccine that empowers the body with anti-tumor immune activity. Further studies are needed to confirm the in situ vaccination accompanied by immune activation.

## 4. Conclusions

Gd_2_O_3_ nanotubes with lengths of 70–80 nm, diameters of 20–30 nm, and pore sizes of up to 18 nm were synthesized by a facile one-pot solvothermal method using gadolinium acetate hydrate, urea, and ethanol. The Gd_2_O_3_ nanotubes were composed of amorphous Gd_2_O_3_ without impurity phases. The Gd_2_O_3_ nanotubes showed good adsorption capacity of OVA and TLR7a, with a loading efficiency of about 100%. The Gd_2_O_3_ nanotubes showed pH-sensitive degradation and biomolecule release from Gd_2_O_3_-OVA and Gd_2_O_3_-TLR7a; the release of gadolinium ions, OVA, and TLR7a was slow in a slightly alkaline environment but fast in an acidic environment. The Gd_2_O_3_ nanotubes showed 2.6–6.0 times higher payload retention around the injection site, 3.1 times higher cellular uptake, 1.7 times higher IL1β secretion, 1.4 times higher TNFα secretion by BMDCs, and markedly enhanced draining lymph node delivery properties. The combination of OVA, TLR7a, and Gd_2_O_3_ nanotubes significantly inhibited tumor growth and increased survival rate compared with only OVA-TLR7a, only OVA, and saline. The combination of OVA, TLR7a, and Gd_2_O_3_ nanotubes significantly increased IFNγ and TNFα expression levels in splenocytes of mice. The Gd_2_O_3_ nanotubes were found to be biocompatible and showed the radiation sensitization effect.

## Data Availability

The data presented in this study are available on request from the corresponding author.

## Supplementary Materials

The following supporting information can be downloaded at: https://www.mdpi.com/article/10.3390/vaccines12040373/s1, Figure S1: The effects of unloaded Gd_2_O_3_ nanotubes and Gd_2_O_3_-OVA on BMDC maturation.

## References

[1] Mellman I., Coukos G., Dranoff G. (2011). Cancer immunotherapy comes of age. Nature.

[2] McNutt M. (2013). Cancer Immunotherapy. Science.

[3] Elmusrati A., Wang J., Wang C.Y. (2021). Tumor microenvironment and immune evasion in head and neck squamous cell carcinoma. Int. J. Oral Sci..

[4] Mascaux C., Angelova M., Vasaturo A., Beane J., Hijazi K., Anthoine G., Buttard B., Rothe F., Willard-Gallo K., Haller A. (2019). Immune evasion before tumour invasion in early lung squamous carcinogenesis. Nature.

[5] Steinman R.M., Banchereau J. (2007). Taking dendritic cells into medicine. Nature.

[6] Banchereau J., Steinman R.M. (1998). Dendritic cells and the control of immunity. Nature.

[7] Sahin U., Tureci O. (2018). Personalized vaccines for cancer immunotherapy. Science.

[8] Schuller S., Wisgrill L., Sadeghi K., Gindl E., Helmer H., Husslein P., Berger A., Spittler A., Forster-Waldl E. (2016). The TLR-specific adjuvants R-848 and CpG-B endorse the immunological reaction of neonatal antigen-presenting cells. Pediatr. Res..

[9] Lynn G.M., Laga R., Darrah P.A., Ishizuka A.S., Balaci A.J., Dulcey A.E., Pechar M., Pola R., Gerner M.Y., Yamamoto A. (2015). In vivo characterization of the physicochemical properties of polymer-linked TLR agonists that enhance vaccine immunogenicity. Nat. Biotechnol..

[10] Soria I., Myhre P., Horton V., Ellefson P., McCarville S., Schmitt K., Owens M. (2000). Effect of food on the pharmacokinetics and bioavailability of oral imiquimod relative to a subcutaneous dose. Int. J. Clin. Pharmacol. Ther..

[11] Stockfleth E., Trefzer U., Garcia-Bartels C., Wegner T., Schmook T., Sterry W. (2003). The use of Toll-like receptor-7 agonist in the treatment of basal cell carcinoma: An overview. Br. J. Dermatol..

[12] Hemmi H., Kaisho T., Takeuchi O., Sato S., Sanjo H., Hoshino K., Horiuchi T., Tomizawa H., Takeda K., Akira S. (2002). Small anti-viral compounds activate immune cells via the TLR7 MyD88-dependent signaling pathway. Nat. Immunol..

[13] Lee J., Chuang T.H., Redecke V., She L.P., Pitha P.M., Carson D.A., Raz E., Cottam H.B. (2003). Molecular basis for the immunostimulatory activity of guanine nucleoside analogs: Activation of Toll-like receptor 7. Proc. Natl. Acad. Sci. USA.

[14] Nguyen T.L., Choi Y., Kim J. (2019). Mesoporous Silica as a Versatile Platform for Cancer Immunotherapy. Adv. Mater..

[15] Li X., Yamazaki T., Ebara M., Shirahata N., Hanagata N. (2023). Nanoengineered coordination polymers boost cancer immunotherapy. Mater. Today.

[16] Wang X.P., Li X., Onuma K., Sogo Y., Ohno T., Ito A. (2013). Zn- and Mg- Containing Tricalcium Phosphates-Based Adjuvants for Cancer Immunotherapy. Sci. Rep..

[17] Wang X.P., Li X., Ito A., Sogo Y., Watanabe Y., Hashimoto K., Yamazaki A., Ohno T., Tsuji N.M. (2018). Synergistic effects of stellated fibrous mesoporous silica and synthetic dsRNA analogues for cancer immunotherapy. Chem. Commun..

[18] Wang X.P., Li X., Ito A., Sogo Y., Ohno T. (2014). Pore-size dependent immunogenic activity of mesoporous silica-based adjuvants in cancer immunotherapy. J. Biomed. Mater. Res. A.

[19] Wang X., Ihara S., Li X., Ito A., Sogo Y., Watanabe Y., Tsuji N.M., Yamazaki A. (2018). Si-doping increases the adjuvant activity of hydroxyapatite nanorods. Colloids Surf. B Biointerfaces.

[20] Nuhn L., De Koker S., Van Lint S., Zhong Z.F., Catani J.P., Combes F., Deswarte K., Li Y.P., Lambrecht B.N., Lienenklaus S. (2018). Nanoparticle-Conjugate TLR7/8 Agonist Localized Immunotherapy Provokes Safe Antitumoral Responses. Adv. Mater..

[21] Yang R., Xu J., Xu L.G., Sun X.Q., Chen Q., Zhao Y.H., Peng R., Liu Z. (2018). Cancer Cell Membrane-Coated Adjuvant Nanoparticles with Mannose Modification for Effective Anticancer Vaccination. ACS Nano.

[22] Shao Y.Z., Tian X.M., Hu W.Y., Zhang Y.Y., Liu H., He H.Q., Shen Y.Y., Xie F.K., Li L. (2012). The properties of Gd_2_O_3_-assembled silica nanocomposite targeted nanoprobes and their application in MRI. Biomaterials.

[23] Kimura Y., Kamisugi R., Narazaki M., Matsuda T., Tabata Y., Toshimitsu A., Kondo T. (2012). Size-controlled and biocompatible Gd_2_O_3_ nanoparticles for dual photoacoustic and MR imaging. Adv. Healthc. Mater..

[24] Kawashima T., Kosaka A., Yan H., Guo Z., Uchiyama R., Fukui R., Kaneko D., Kumagai Y., You D.J., Carreras J. (2013). Double-stranded RNA of intestinal commensal but not pathogenic bacteria triggers production of protective interferon-beta. Immunity.

[25] Li X., Wang X.P., Ito A., Tsuji N.M. (2020). A nanoscale metal organic frameworks-based vaccine synergises with PD-1 blockade to potentiate anti-tumour immunity. Nat. Commun..

[26] Kapadia C.H., Tian S.M., Perry J.L., Sailer D., Luft J.C., DeSimone J.M. (2018). Extending antigen release from particulate vaccines results in enhanced antitumor immune response. J. Control. Release.

[27] Jewell C.M., Lopez S.C.B., Irvine D.J. (2011). In situ engineering of the lymph node microenvironment via intranodal injection of adjuvant-releasing polymer particles. Proc. Natl. Acad. Sci. USA.

[28] Johansen P., Storni T., Rettig L., Qiu Z.Y., Der-Sarkissian A., Smith K.A., Manolova V., Lang K.S., Senti G., Mullhaupt B. (2008). Antigen kinetics determines immune reactivity. Proc. Natl. Acad. Sci. USA.

[29] de Titta A., Ballester M., Julier Z., Nembrini C., Jeanbart L., van der Vlies A.J., Swartz M.A., Hubbell J.A. (2013). Nanoparticle conjugation of CpG enhances adjuvancy for cellular immunity and memory recall at low dose. Proc. Natl. Acad. Sci. USA.

[30] Murtaugh M.P., Foss D.L. (2002). Inflammatory cytokines and antigen presenting cell activation. Vet. Immunol. Immunopathol..

[31] Soto J.A., Galvez N.M.S., Andrade C.A., Pacheco G.A., Bohmwald K., Berrios R.V., Bueno S.M., Kalergis A.M. (2020). The Role of Dendritic Cells during Infections Caused by Highly Prevalent Viruses. Front. Immunol..

[32] Martin-Gayo E., Yu X.G. (2019). Role of Dendritic Cells in Natural Immune Control of HIV-1 Infection. Front. Immunol..

[33] Wang H., Mooney D.J. (2018). Biomaterial-assisted targeted modulation of immune cells in cancer treatment. Nat. Mater..

[34] Irvine D.J., Swartz M.A., Szeto G.L. (2013). Engineering synthetic vaccines using cues from natural immunity. Nat. Mater..

[35] Phuengkham H., Song C., Um S.H., Lim Y.T. (2018). Implantable Synthetic Immune Niche for Spatiotemporal Modulation of Tumor-Derived Immunosuppression and Systemic Antitumor Immunity: Postoperative Immunotherapy. Adv. Mater..

[36] Waldman A.D., Fritz J.M., Lenardo M.J. (2020). A guide to cancer immunotherapy: From T cell basic science to clinical practice. Nat. Rev. Immunol..

[37] Kim H., Niu L., Larson P., Kucaba T.A., Murphy K.A., James B.R., Ferguson D.M., Griffith T.S., Panyam J. (2018). Polymeric nanoparticles encapsulating novel TLR7/8 agonists as immunostimulatory adjuvants for enhanced cancer immunotherapy. Biomaterials.

[38] Iwasaki A., Medzhitov R. (2004). Toll-like receptor control of the adaptive immune responses. Nat. Immunol..

[39] Gocher A.M., Workman C.J., Vignali D.A.A. (2022). Interferon-gamma: Teammate or opponent in the tumour microenvironment?. Nat. Rev. Immunol..

[40] Jorgovanovic D., Song M., Wang L., Zhang Y. (2020). Roles of IFN-gamma in tumor progression and regression: A review. Biomark. Res..

[41] van Horssen R., Hagen T.L.M.T., Eggermont A.M.M. (2006). TNF-alpha in cancer treatment: Molecular insights, antitumor effects, and clinical utility. Oncologist.

[42] Curnis F., Sacchi A., Borgna L., Magni F., Gasparri A., Corti A. (2000). Enhancement of tumor necrosis factor alpha antitumor immunotherapeutic properties by targeted delivery to aminopeptidase N (CD13). Nat. Biotechnol..

[43] Kreiter S., Vormehr M., van de Roemer N., Diken M., Lower M., Diekmann J., Boegel S., Schrors B., Vascotto F., Castle J.C. (2015). Mutant MHC class II epitopes drive therapeutic immune responses to cancer. Nature.

[44] Balachandran V.P., Rojas L.A., Sethna Z., Soares K., Derhovanessian E., Mueller F., Yadav M., Basturk O., Gonen M., Wei A.C.C. (2022). Phase I trial of adjuvant autogene cevumeran, an individualized mRNA neoantigen vaccine, for pancreatic ductal adenocarcinoma. J. Clin. Oncol..

