# Supplementary material for: TLR7 Agonist-Loaded Gadolinium Oxide Nanotubes Promote Anti-Tumor Immunity by Activation of Innate and Adaptive Immune Responses
Source: Vaccines (Basel). 2024 Apr 1;12(4):373. doi: 10.3390/vaccines12040373 (PMC11053986; doi:10.3390/vaccines12040373)
Supplement: Supplementary file 1 [file vaccines-12-00373-s001.zip › vaccines-2917094-supplementary.pdf]

Supporting Information

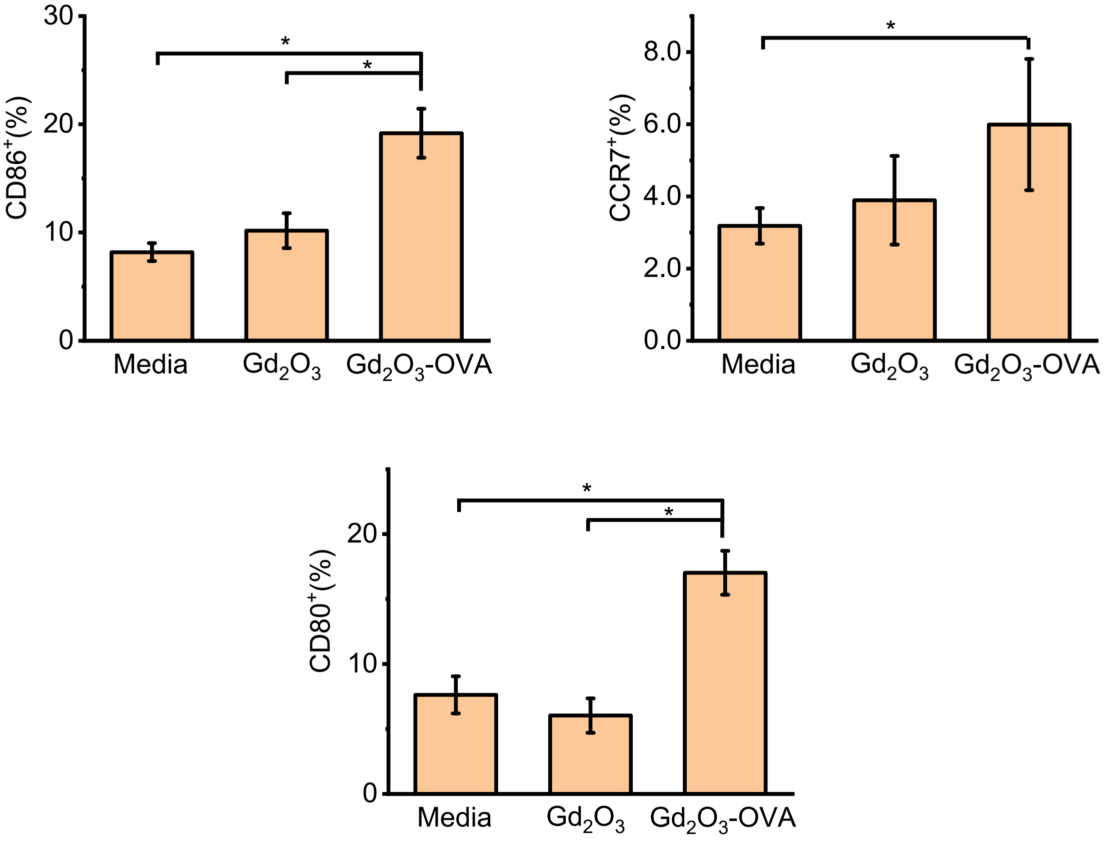

Figure S1. The effects of unloaded Gd_2O_3 nanotubes and Gd_2O_3-OVA on BMDC maturation
